# Characterization of Patients Unsuited for Transcatheter Mitral Valve Interventions

**DOI:** 10.3390/jcm14207275

**Published:** 2025-10-15

**Authors:** Carolina Göttsche Esperança Clara, Hannah Eustergerling, Johanna Isabella Pepping, Vanessa Trenkpohl, Kai Peter Friedrichs, Maria Ivannikova, Tanja Katharina Rudolph, Johanna Bormann, Johannes Kirchner, Max Potratz, Volker Rudolph, Mohammad Kassar, Muhammed Gerçek, Felix Rudolph

**Affiliations:** 1 Clinic for General and Interventional Cardiology/Angiology, Herz- und Diabeteszentrum NRW, Ruhr-Universität Bochum, 32545 Bad Oeynhausen, Germany; 2 Clinic for General and Interventional Cardiology/Angiology, Herz- und Diabeteszentrum NRW, Medizinische Fakultät OWL der Universität Bielefeld, 32545 Bad Oeynhausen, Germany; 3 Universitätsklinik für Kardiologie, Inselspital, 3010 Bern, Switzerland; 4 Feinberg School of Medicine, Northwestern University, Chicago, IL 60208, USA

**Keywords:** mitral regurgitation, transcatheter edge-to-edge-repair, transcatheter mitral valve replacement, mitral valve quantification

## Abstract

**Background/Objectives**: The objective of this study was to characterize echocardiographic characteristics comparing patients accepted or deemed unsuitable for transcatheter mitral valve interventions. **Methods**: We performed a retrospective analysis of 293 patients with severe mitral regurgitation evaluated for mitral transcatheter edge-to-edge repair (M-TEER) or transcatheter mitral valve replacement (TMVR), if patients were primarily identified as unsuitable candidates for M-TEER, at our institution between 2018 and 2023. All patients underwent transthoracic and transesophageal echocardiography, and mitral valve quantification (MVQ) analysis was performed. Feasibility was determined by an interdisciplinary Heart Team based on quantitative data and semi-quantitative echocardiographic parameters, including mean pressure gradient, regurgitation volumes, and morphological aspects alongside clinical assessment. Patient characteristics were evaluated using clinical records and echocardiographic data. **Results**: We screened 195 patients for M-TEER, 168 of which were accepted for the procedure. M-TEER-rejected patients presented with higher regurgitation volumes and higher transmitral pressure gradients than those accepted to undergo M-TEER. We then screened 104 patients for TMVR, and 27 were approved for the procedure. Patients rejected for TMVR presented with lower tenting volume, area, and height and had smaller ventricular diameters. Further, mitral valve area appears to be an important parameter in determining MR treatment strategy. **Conclusions:** The majority of MR patients screened for transcatheter intervention were suitable for M-TEER. However, elevated MPG and more pronounced billowing were the main factors associated with M-TEER exclusion. Conversely, the only morphological parameter associated with TMVR refusal was small left ventricular size. Importantly, the multimorbidity of patients and level of critical illness did not prohibit TMVR.

## 1. Introduction

Mitral valve regurgitation remains the second most common valvular heart disease in the Western World [1]. Mitral regurgitation (MR) is categorized according to etiology in degenerative (primary) MR (DMR) and functional (secondary) MR (FMR) and is traditionally diagnosed using transesophageal echocardiography (TEE). However recent studies have suggested that transthoracic (TTE) may be a feasible alternative for characterizing mitral valve anatomy [2,3].

Untreated MR often leads to worsening of heart failure, atrial fibrillation and pulmonary artery hypertension, and ultimately death [4]. Studies have shown that MR is associated with excess mortality, regardless of coexisting risk factors or etiology. Despite the poor prognosis, only a small percentage of patients, as low as 15%, undergo mitral valve surgery [5].

As MR is associated with age and underlying cardiac pathologies, affected patients are often at elevated surgical risk. For these patients, European guidelines issued a class IIb recommendation for mitral transcatheter edge-to-edge repair (M-TEER) in DMR, and a class IIa recommendation for FMR. More recent trials like the “Mitral vAlve reconsTrucTion for advancEd Insufficiency of Functional or iscHemic ORigiN” (MATTERHORN) and “A Clinical Evaluation of the Safety and Effectiveness of the MitraClip System in the Treatment of Clinically Significant Functional Mitral Regurgitation” (Reshape-HF2) trials have demonstrated the non-inferiority of M-TEER to conventional surgery, even suggesting a possible benefit in regard to safety and less severe MR [6,7].

While most patients are anatomically suited to undergo M-TEER, there is still a sizeable population ineligible for this approach [8]. These patients are then often evaluated for transcatheter mitral valve replacement (TMVR) strategies, including CE-marked devices like the TENDYNE prothesis (Abbott Vascular, Indianapolis), which has shown promising results [9]. While increasing options in transcatheter approaches for MR allow for a larger proportion of patients to receive transcatheter treatment, clinical and anatomical characteristics of patients unsuited for any currently available approach are not yet well described. Hence, we aimed to characterize echocardiographic characteristics in a cohort of patients evaluated for M-TEER and TMVR stratified by eligibility for the procedure as determined by an interdisciplinary heart team.

## 2. Materials and Methods

All consecutive patients that were evaluated for M-TEER eligibility between August 2018 and May 2021, as well as feasibility of TMVR (between January 2020 and January 2023) at the Heart and Diabetes Center NRW, a high-volume tertiary center for valvular heart disease in Germany, were included in this retrospective analysis. Exclusion criteria included pediatric patients (<18 years), patients that did not give written consent or were unable to do so, and asymptomatic patients under medical treatment. Initially, all patients were evaluated for M-TEER, with additional screening for TMVR if M-TEER appeared difficult or not feasible as determined by an interdisciplinary heart team on site. The endpoint of this study was the decision made by the heart team, independently of succeeding treatment and its respective success. Treatment success was defined abiding by Mitral Valve Academic Research Consortium (MVARC) recommendations [10]: technical success (defined as “at exit catheter lab: alive AND successful deployment without detachment and retrieval of the device delivery system AND no emergency surgery”) and device success at 30 days (defined as technical success, mitral regurgitation grade <=2+, alive without stroke, no unplanned surgical or interventional procedures, and no device failure).

Clinical and echocardiographic characteristics were descriptively compared between accepted and refused patients for both M-TEER and TMVR.

Echocardiographic data was obtained from both TTE and TEE. All examinations utilized commercially available equipment, specifically the Vivid 7, E9, and E95 machines (GE Healthcare, Horten, Norway). All imaging was stored centrally and retrospectively analyzed using EchoPAC (GE Healthcare, Chicago, IL, USA, EchoPAC Version 202). Mitral Valve Quantification (MVQ) analysis was performed retrospectively on these images [11]. The study was approved by the Ethics Committee of the Medical Faculty of Ruhr University Bochum and all patients participating in the study gave informed consent.

The statistical analysis was performed using R in RStudio (Version 2024.12.1.563, R. Posit Software, PBC, Boston, MA, USA). The Shapiro–Wilk Test was used to determine whether data was distributed normally. Continuous parametric data was portrayed as mean ± standard deviation (SD), while non-parametric data was displayed as median and interquartile range (IQR). Categorical variables were presented using frequencies and percentages. Comparison between groups were calculated using the Wilcoxon rank test or the Kruskal–Wallis test. Group comparison of categorical variables was performed using Fisher’s exact test and Pearson’s chi-square test. Linear regression analyses were performed to identify parameters associated with ineligibility for either intervention (i.e., who had to remain under conservative management and those who received surgery) within the whole cohort, for which the Odds Ratio (OR), alongside the 95% confidence interval (CI), was indicated. A *p*-value < 0.05 was considered statistically significant.

## 3. Results

### 3.1. Patient Population

**Figure 1:**
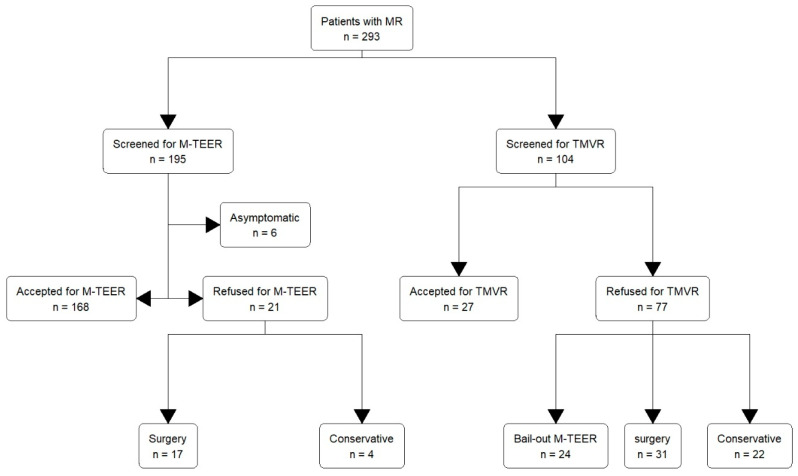
Flow chart showing heart team decision regarding mitral valve regurgitation therapy. MR = mitral Regurgitation; M-TEER = mitral transcatheter edge-to-edge repair; TMVR = transcatheter mitral valve replacement.

A total of 293 patients were included in this analysis. Of these, 195 (66.6%) were screened to undergo M-TEER and 104 (43.5%) were evaluated for TMVR. Six patients (3.1%) were excluded from the study due to low symptomatic burden, 168 patients (86.2%) were accepted (M-TEER-A), and 21 patients (10.8%) were refused (M-TEER-R) M-TEER. In the cohort of the M-TEER-R patients, seventeen (81.0%) ultimately underwent surgery and four (19.0%) remained under conservative management. Among the patients evaluated for TMVR, 27 (26.0%) were accepted (TMVR-A) and 77 (74.0%) refused (TMVR-R). Of those patients refused TMVR, 24 (32.4%) underwent bail-out M-TEER, 31 (40.3%) underwent surgery, and 22 (28.6%) remained under conservative treatment. Hence, 26 (8.8%) of all patients were excluded from any intervention or surgery and remained under conservative treatment. The heart team decision path is portrayed in Figure 1. Regarding the procedural success we found, MVARC-defined technical success was achieved in 96% of patients receiving primary M-TEER, 89% of patients receiving bail-out M-TEER, 96% of TMVR patients, and 100% of surgical patients. MVARC-defined device success at 30 days was achieved in 90% of M-TEER patients, 65% of bail-out M-TEER patients, and 73% of TMVR patients. There is limited data available for the surgical patients.

### 3.2. Group Comparison of M-TEER Accepted and Refused Patients

M-TEER-A patients were significantly older, at a median age of 80 years [IQR 74–83] versus 75 years [IQR 70–79] for M-TEER-R patients (*p* = 0.021). Gender distribution was 44% and 29% female in the M-TEER-A and M-TEER-R groups, respectively (*p* = 0.20). M-TEER-A patients had a mean Body Mass Index (BMI) of 26.4 ± 5.7 kg/m^2^, while M-TEER-R patients had a mean BMI of 27.4 ± 8.0 kg/m^2^ (*p* = 0.50) (Table 1).

MR etiology differed significantly, with lower rates of DMR in the M-TEER-A group, at 33% versus 57% in the M-TEER-R patients (*p* = 0.002), while MR severity appeared to be comparable between the groups (*p* = 0.20). In total, 81% of M-TEER-A patients presented as New York Heart Association (NYHA) class ≥ III, versus 86% in the M-TEER-R group (*p* = 0.50).

**Figure 2:**
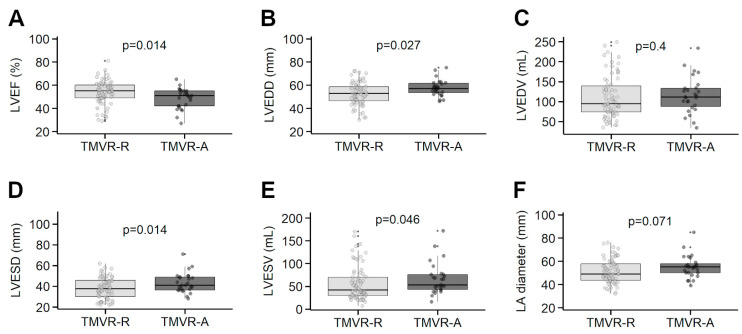
Boxplots for selected echocardiographic parameters between patients accepted (-A) and refused (-R) for transcatheter mitral valve replacement (TMVR). LVEF = left ventricular ejection fraction (Panel **A**); LVEDD = left ventricular end-diastolic diameter (Panel **B**); LVEDV = left ventricular end-diastolic volume (Panel **C**); LVESD = left ventricular end-systolic diameter (Panel **D**); LVESV = left ventricular end-systolic volume (Panel **E**); LA = left atrium (Panel **F**).

Mean left ventricular ejection fraction (LVEF) was 57 ± 16% in the M-TEER-A group and 50 ± 10% in the M-TEER-R group (*p* = 0.040). The median left atrium (LA) diameter was 52 mm [IQR 46–59] in the M-TEER-A group versus 48 mm [IQR 39–55] in the M-TEER-R cohort (*p* = 0.048). The MPG differed significantly in the M-TEER-A group (2.2 ± 1.1 mmHg) versus in M-TEER-R patients (4.0 ± 1.5 mmHg) (*p* < 0.001) (Figure 2).

**Figure 3:**
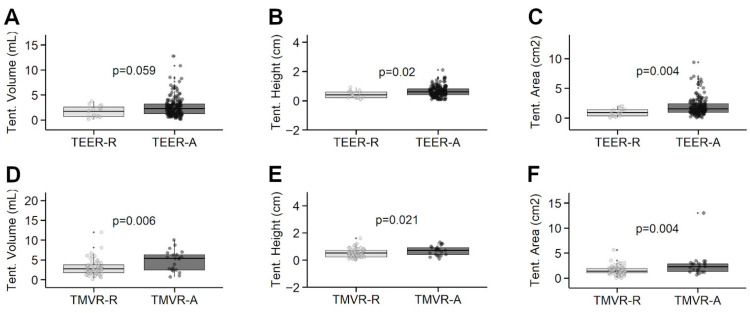
Boxplots for selected parameters derived from mitral quantification analysis between patients accepted (-A) and refused (-R) for mitral transcatheter edge-to-edge repair (M-TEER) or transcatheter mitral valve replacement (TMVR). Tent. = tenting. Tenting Volume in M-TEER (Panel **A**) and TMVR (Panel **D**); Tenting Height in M-TEER (Panel **B**) and TMVR (Panel **E**); Tenting Area in M-TEER (Panel **C**) and TMVR (Panel **F**).

Notable differences in the MVQ analyses included higher mean tenting height in the M-TEER-A patients (0.6 ± 0.4 cm) compared to tenting height in those rejected (0.4 ± 0.2 cm) (*p* = 0.020) (Table 2). Similarly, the mean tenting area was higher at 1.9 ± 1.5 cm^2^ among M_TEER-A versus 1.0 ± 0.7 cm^2^ in the M-TEER-R patients (*p* = 0.004). Additionally, mean anterior and posterior billowing heights were 0.1 ± 0.4 mm and 1.0 ± 1.8 mm, respectively, in the M-TEER-A cohort and 0.6 ± 1.0 mm and 2.2 ± 2.7 mm in the M-TEER-R group (*p* < 0.001 and *p* = 0.023, respectively) (Figure 3).

### 3.3. Group Comparisons of TMVR Accepted and Refused Patients

The median age of TMVR-A patients was 79 years [IQR 72–83] and 80 years [IQR 77–83] in the TMVR-R group (*p* = 0.70). While 41% of the TMVR-A patients were female, 53% were female in the TMVR-R group (*p* = 0.30).

MR etiology was DMR in 41% of TMVR-A patients and 48% of patients in the TMVR-R group (*p* = 0.20). MR severity also appeared to be comparable (*p* = 0.70). Functional status as assessed by NYHA class appeared to be comparable, with 78% presenting as NYHA ≥ III in the accepted and 84% in the rejected group (*p* = 0.60) (Table 3).

The TMVR-A group presented with a mean LVEF of 49 ± 9% versus 54 ± 10% in the TMVR-R group (*p* = 0.014). The median left ventricular end-diastolic diameter (LVEDD) was 57 mm [IQR 53–62] in the TMVR-A group and 53 mm [IQR 46–59] in the TMVR-R group (*p* = 0.027) (Table 4). The median left ventricular end-systolic diameter (LVESD) and end-systolic volume (LVESV) were 41 mm [IQR 36–50] and 53 mL [IQR 42–76] in the TMVR-A group and 37 mm [IQR 30–46] and 42 mL [IQR 29–70], respectively, in the TMVR-R group (*p* = 0.014 and *p* = 0.046, Figure 2).

Notable findings in the MVQ analyses included significantly higher tenting height in the TMVR-A patients (0.7 ± 0.4 cm) compared to those in the TMVR-R cohort (0.5 ± 0.3 cm, *p* = 0.021). The mean tenting area was 2.6 ± 2.5 cm^2^ in the TMVR-A and 1.5 ± 0.9 cm^2^ in the TMVR-R group (*p* = 0.004), while tenting volume was 5.5 ± 4.2 mL in the TMVR-A patients, as opposed to 3.4 ± 3.1 mL in the TMVR-R group (*p* = 0.006, Figure 3).

### 3.4. Predictors of Ineligibility for Any Intervention

**Figure 4:**
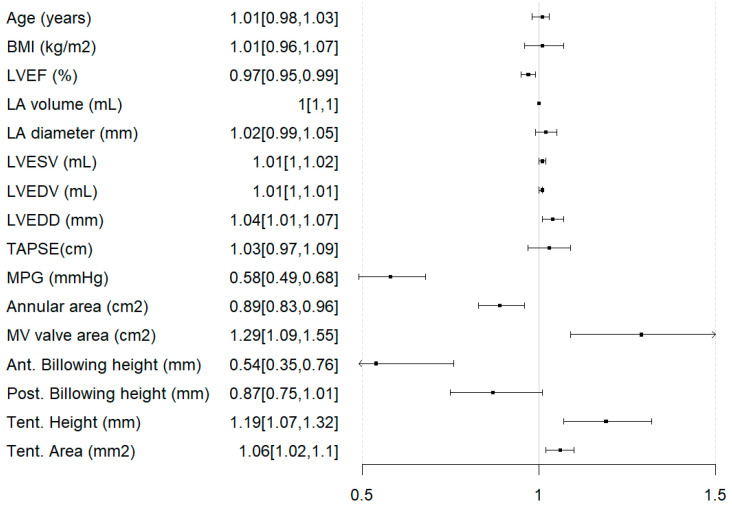
Forest plot for parameters predicting rejection of both transcatheter edge-to-edge repair and transcatheter mitral valve replacement. BMI = Body Mass Index; LVEF = left ventricular ejection fraction; LA = left atrium; LVESV = left ventricular end-systolic volume; LVEDV = left ventricular end-diastolic volume; LVEDD = left ventricular end-diastolic diameter; TAPSE = tricuspid annular plane systolic excursion; MPG = mean pressure gradient (across the mitral valve); MV area = mitral valve area; Ant. = anterior; Post. = posterior; Tent. = tenting.

Linear regression analysis revealed LVEF (OR = 0.97 [95% CI: 0.95–0.99]), MPG (OR = 0.58 [95% CI: 0.49–0.68]), anterior billowing height (OR = 0.54 [95% CI: 0.35–0.76]), and smaller annular area (OR = 0.58 [95% CI: 0.89–0.96]) to be associated with higher odds of being excluded from intervention. Conversely, mitral valve area (OR = 1.29 [95% CI: 1.09–1.55]), LVEDD (OR = 1.04 [95% CI: 1.01–1.07]), LVESV (OR = 1.01 [95% CI: 1.00–1.02]), LVEDV (OR = 1.01 [95% CI: 1.00–1.01]), tenting height, and area were associated with lower odds (OR = 1.19 [95% CI: 1.07–1.32] and OR = 1.06 [95% CI: 1.02–1.1], respectively, Figure 4). Planimetric mitral valve area and MPG were significantly correlated (*r* = −0.225, *p* < 0.001).

**Figure 5:**
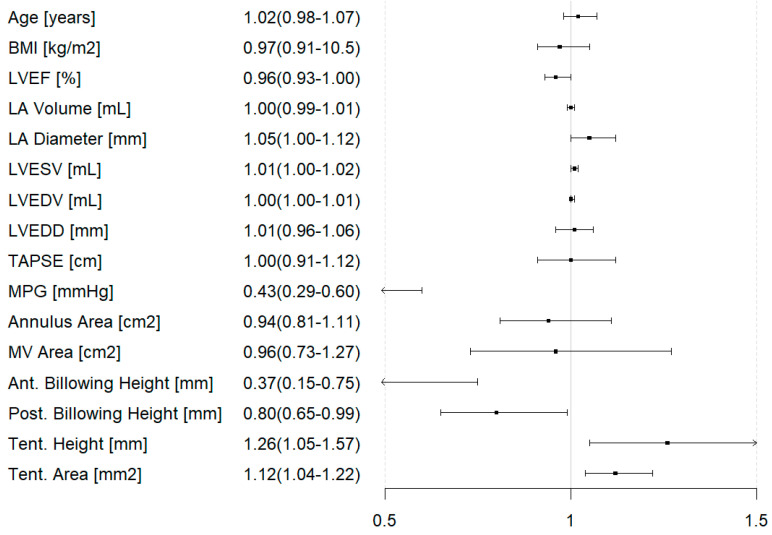
Forest plot for parameters predicting rejection of transcatheter edge-to-edge repair. BMI = Body Mass Index; LVEF = left ventricular ejection fraction; LA = left atrium; LVESV = left ventricular end-systolic volume; LVEDV = left ventricular end-diastolic volume; LVEDD = left ventricular end-diastolic diameter; TAPSE = tricuspid annular plane systolic excursion; MPG = mean pressure gradient (across the mitral valve); MV area = mitral valve area; Ant. = anterior; Post. = posterior; Tent. = tenting.

When looking at drivers of M-TEER rejection, linear regression analysis revealed LVEF (OR = 0.96 [95% CI: 0.93–1.00]), anterior billowing height (OR= 0.37 [95% CI: 0.15–0.75]), posterior billowing height (OR = 0.80 [95% CI: 0.65–0.99]), LA diameter (OR = 1.05 [95% CI: 1.00–1.12]), MPG (OR = 0.43 [95% CI: 0.29–0.60]), tenting height (OR = 1.26 [95% CI:1.05–1.57]), and tenting area (OR = 1.12 [95% CI: 1.04–1.22]) (Figure 5).

**Figure 6:**
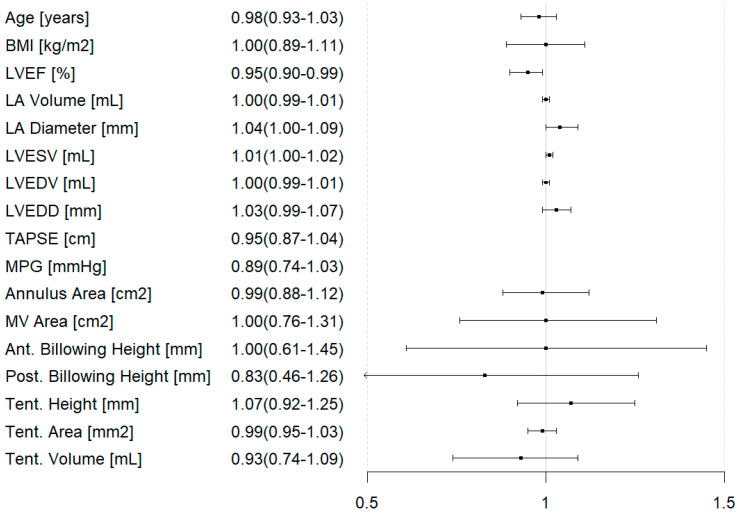
Forest plot for parameters predicting rejection of transcatheter mitral valve replacement. BMI = Body Mass Index; LVEF = left ventricular ejection fraction; LA = left atrium; LVESV = left ventricular end-systolic volume; LVEDV = left ventricular end-diastolic volume; LVEDD = left ventricular end-diastolic diameter; TAPSE = tricuspid annular plane systolic excursion; MPG = mean pressure gradient (across the mitral valve); MV area = mitral valve area; Ant. = anterior; Post. = posterior; Tent. = tenting.

Conversely, LVEF was the only parameter that appeared to influence TMVR rejection (OR = 0.95 [95% CI: 0.90–0.99]) (Figure 6).

When comparing the two cohorts screened for M-TEER and TMVR, respectively, patients screened for TMVR presented with a higher surgical risk score (EuroSCORE II 5.1% [IQR 3.1–9.1] versus 4.3% [IQR 2.5–7.3], *p* = 0.049) and more often had a history of previous cardiac surgery: 80% versus 22% of M-TEER-screened patients (*p* < 0.001) (Table 5). Echocardiographic parameters also differed, as the TMVR-screened patients appeared to have a higher transmitral mean pressure gradient (MPG) (4.6 ± 3 mmHg vs. 2.4 ± 1 mmHg, *p* < 0.001), as well as a smaller mitral valve orifice area (3.6 ± 2 cm^2^ vs. 4.8 ± 2 cm^2^, *p* < 0.001) and larger annuli (annular area 15 ± 4 cm^2^ vs. 12 ± 3 cm^2^, *p <* 0.001) (Table 6).

## 4. Discussion

### 4.1. Identification of Suitable Transcatheter Mitral Valve Intervention Methods According to Patient Characteristics

In this retrospective observational study, we characterize clinical and echocardiographic parameters of patients accepted and refused for M-TEER and TMVR. We found higher and more advanced MPG within those patients rejected for M-TEER. Elevated baseline MPG imposes a higher risk of developing relevant mitral stenosis after implantation of one or multiple clips needed to address MR, explaining this observation [12]. Currently, no randomized controlled trial evaluated the safety and efficacy of M-TEER for primary mitral regurgitation. Observational data revealed favorable outcomes even in complex degenerative MR [13,14]. As our understanding of M-TEER for addressing primary MR has developed in recent years, this might introduce a selection bias opposing primary MR—and hence those patients with more advanced billowing—within this analysis.

TMVR-accepted patients were more likely to exhibit echocardiographic parameters surrogating larger or more dilated left ventricles, including more extensive tenting, indicative of the necessity for sufficient space to fit a TMVR prothesis without risk of left ventricular outflow tract obstruction (LVOTO) [15]. This is further supported by our linear regression analysis, which suggests that a smaller annular size is a factor associated with TMVR rejection. Although there are plenty of experimental devices in trial, TMVR devices are still limited in size, due to possible interference with nearby anatomical structures, ranging from the left ventricular outflow tract to the circumflex coronary artery to the coronary sinus. Further, when aiming for a transseptal approach, larger prostheses requiring larger delivery devices become increasingly problematic.

Moreover, the aortomitral angle was found to be larger in the TMVR-accepted group. Studies on systolic anterior motion (SAM) of the mitral valve have shown an association between lower aortomitral angle and SAM—a feared complication of TMVR in which the left ventricular outflow is obstructed. In contrast, a greater aortomitral angle is associated with more efficient heart motion, indicating that fear of LVOTO is once more a driver in TMVR patient selection [16]. Nevertheless, innovative procedures like balloon-assisted translocation of the anterior mitral leaflet (BATMAN) and Laceration of the Anterior Mitral Leaflet to Prevent Outflow Obstruction (LAMPOON) are being developed to avoid this feared complication [17,18].

Our regression analysis revealed larger LV anatomies (LVESV, LVEDV, and LVEDD), tenting height, and area to be associated with a higher likelihood of receiving intervention. Accordingly, patients with smaller annuli were more likely to be excluded from intervention, as TMVR is prohibited due to the limited available space and M-TEER also imposes the risk of relevant stenosis after the procedure. While higher MPG was found to be a predictor of remaining under conservative treatment across the whole cohort, this was likely driven by the patients evaluated for M-TEER only, as MPG alone does not prohibit TMVR. Additionally, calcification in the grasping area and a short posterior leaflet might also result in M-TEER procedural failure [19,20]. Conversely, the TENDYNE prothesis was designed to address severe mitral annular calcification and was recently approved for treatment of mitral regurgitation and stenosis in the US. Even though patients evaluated for TMVR were more ill and exhibited signs of more advanced MR in comparison to those evaluated for M-TEER, remarkably, patient frailty was not a limiting factor in receiving TMVR. Further, small left ventricular size was the only morphological parameter to predict rejection from TMVR. Recent evidence suggests that a narrow antero-posterior thoracic diameter measured by TTE might assist in identifying patients with smaller ventricular chambers, which, given our findings, might provide additional clinical value in the evaluation of transcatheter approaches for MR [21,22].

Even though inclusion criteria for TMVR and M-TEER treatment have broadened in the past years, a significant proportion of patients are still rejected from either treatment and exposed to a high symptomatic burden and, more importantly, excess mortality. Subsequently this is the patient cohort that needs to be meticulously studied, as the prevalence of valvular heart disease is expected to rise in the coming decades, leading to a larger patient population with unmet treatment needs [23].

### 4.2. Clinical Implications

First of all, M-TEER is a highly safe and effective treatment for mitral regurgitation, and the majority of patients can be treated successfully. However, despite the large proportion of suitable candidates for M-TEER, a “one-size-fits-all” strategy cannot be optimal for every case of MR, particularly in the presence of valvular or annular calcification. Even in the management of aortic stenosis, multiple device options are required to address anatomical variability. The TENDYNE device was introduced over 10 years ago, and the patient cohort benefitting from this device has been ever-expanding since [24]. Even though there are still significant rates of complications, the results are promising, especially when looking at the rise in transcatheter aortic valve replacement (TAVR) over the last two decades. TAVR now provides a safe treatment option for patients with severe aortic stenosis who were too frail for surgery, which is performed frequently in many hospitals. Initially, procedural complications were high; however, as newer devices have developed and experience with these devices has increased, complication- and intervention-related mortality has decreased [25]. Similarly, TMVR is gaining traction, and a multitude of different devices apart from TENDYNE are currently undergoing testing. However, there are several differences to be noted between TAVR and TMVR. For one, the mitral valve annulus is often larger and more asymmetrical, leading to a higher rate of paravalvular leakage (PVL) [26]. Additionally, as opposed to the aortic valve, there is often a lack of annular calcification to provide proper anchoring to the prothesis. One of the most feared complications of TMVR is LVOTO, since the protheses are often large due to their anchoring systems, which can interact with the subvalvular structures. Moreover, as hinted at above, there are other anatomical structures in the proximity that need to be preserved in TMVR, such as the coronary sinus and the circumflex artery [15].

Furthermore, one of several aspects that have led to the popularity of TAVR is implantation through a transfemoral access site, which is possible in most patients. The most common access site in currently available TMVR systems is a transapical approach, which is more invasive by nature and interacts with the subvalvular apparatus and LV myocardium. The most promising approach is the transseptal one, which is the method used in M-TEER. However, in contrast to M-TEER systems, TMVR systems have delivery devices of a bigger diameter. This increases the chances of complications, including iatrogenic atrial septum defects [27,28].

While the development of additional transcatheter approaches is essential to enable treatment for as many patients as possible and to select the most suitable approach for each individual, these alternative approaches may struggle to gain acceptance in clinical practice due to the overwhelming success of M-TEER, which is widely adopted and can be performed quickly and effectively in a large proportion of patients already. Nonetheless, patients deemed unsuitable for M-TEER due to complex anatomy and mitral leaflet calcifications profit from TMVR immensely.

### 4.3. Study Limitations

Several limitations apply to this study. First, this study was designed as a retrospective, single-center, observation-only study, and the observed collective is rather small. Second, we only included patients discussed in our interdisciplinary heart team, which might introduce a selection bias. Third, while all patients exhibited characteristics that rectified the evaluation of transcatheter approaches, a sizable portion underwent surgery after transcatheter approaches were deemed ineligible, which might be associated with elevated risk in these patients. While the endpoint of our regression analysis was those patients who had to remain under conservative treatment or received surgery (i.e., excluded from either transcatheter approach), which might introduce some bias to this analysis, we chose this endpoint for its clinical relevance. Lastly, the aim of this study was to identify parameters associated with acceptance or refusal of M-TEER and/or TMVR; we did not investigate possible associations with procedural success and medical follow-up.

## 5. Conclusions

In this retrospective analysis, we found that the majority of patients with severe mitral regurgitation were suitable for M-TEER. Exclusion from M-TEER was primarily associated with elevated mean pressure gradients and more pronounced mitral valve billowing. In contrast, for patients subsequently evaluated for TMVR, only a smaller left ventricular size predicted rejection. Both insights confirm current clinical decision-making processes and thereby strengthen our understanding of existing decision recommendations. Importantly, patient multimorbidity and critical illness did not preclude eligibility for TMVR.

## Figures and Tables

**Table 1:** Baseline demographic and clinical characteristics among patients evaluated for M-TEER.

Characteristic	M-TEER Refused	M-TEER Accepted	*p*-Value ^1^
	*n* = 21	*n* = 168	
Age, years [median (IQR)]	75 (70–79)	80 (74–83)	0.02
Female [*n* (%)]	6 (29)	74 (44)	0.20
BMI, kg/m^2^ [mean ± SD]	27.4 ± 8.0	26.4 ± 5.7	0.50
STS Score, % [median (IQR)]	1.3 (0.8–2.7)	2.6 (1.5–4.6)	0.016
EuroSCORE II, % [median (IQR)]	2.3 (1.5–3.5)	4.5 (2.7–7.4)	0.004
Atrial fibrillation [*n* (%)]	15 (71)	109 (65)	0.60
Prior MI [*n* (%)]	4 (19)	27 (16)	0.80
Prior cardiac surgery [*n* (%)]	4 (19)	38 (23)	>0.90
NYHA class baseline ≥3 [*n* (%)]	17 (81)	144 (86)	0.50
Prior MV intervention			0.003
None [*n* (%)]	17 (81)	165 (98)	
Prior MV replacement [*n* (%)]	2 (9.5)	0 (0)	
Prior MV repair [*n* (%)]	2 (9.5)	3 (2)	
MV disease			0.20
MV regurgitation [*n* (%)]	20 (90)	161 (96)	
Combined MV disease [*n* (%)]	1 (0.1)	7 (0.04)	
MV Stenosis [*n* (%)]	0 (0)	0 (0)	
MV regurgitation etiology			0.002
Degenerative [*n* (%)]	12 (57)	56 (33)	
Functional [*n* (%)]	4 (19)	96 (57)	
Mixed [*n* (%)]	5 (24)	16 (10)	
Residual [*n* (%)]	0 (0)	0 (0)	
MR severity			0.20
Mild [*n* (%)]	1 (5)	0 (0)	
Mild to moderate [*n* (%)]	0 (0)	5 (3)	
Moderate to severe [*n* (%)]	9 (43)	81 (48)	
Severe [*n* (%)]	11 (52)	82 (49)	

Table Legend: BMI = Body Mass Index; EuroSCORE II = European System for Cardiac Operative Risk Evaluation Score II; IQR = interquartile range; MI = myocardial infarction; MR = mitral regurgitation; M-TEER = mitral transcatheter edge-to-edge repair; MV = mitral valve; NYHA = New York Heart Association; STS Score = Society of Thoracic Surgeons Risk Score. ^1^ Wilcoxon rank test, Kruskal–Wallis test, Fisher’s exact test, or Pearson’s chi-square test, as appropriate.

**Table 2:** Baseline echocardiographic characteristics among patients evaluated for M-TEER.

Characteristic	M-TEER Refused	M-TEER Accepted	*p*-Value ^1^
	*n* = 21	*n* = 168	
LVEF, % [mean ± SD]	50 ± 15	57 ± 16	0.04
LVEDD, mm [median (IQR)]	56 (51–61)	57 (51–63)	0.70
LVEDV, ml [median (IQR)]	113 (84–143)	100 (71–158)	0.80
LVESD, mm [median (IQR)]	36 (32–44)	39 (33–50)	0.30
LVESV, ml [median (IQR)]	48 (23–69)	47 (31–87)	0.40
LA Diameter, mm [median (IQR)]	48 (39–55)	52 (46–59)	0.048
LA Volume, mL [mean ± SD]	131 ± 99	127 ± 60	0.30
RA Area, cm^2^ [mean ± SD]	21 ± 10	24 ± 10	0.14
RV Basal Diameter, mm [mean ± SD]	33 ± 10	38 ± 10	0.088
TAPSE, mm [mean ± SD]	19 ± 4	19 ± 5	0.90
MR EROA, cm^2^ [mean ± SD]	0.4 ± 0.3	0.4 ± 0.3	0.60
MR Volume, ml [mean ± SD]	58 ± 28	58 ± 34	0.70
MR V_max_, m/s [mean ± SD]	6 ± 1	5 ± 1	0.006
MR EROA (TEE), mm [mean ± SD]	0.4 ± 0.2	0.4 ± 0.3	0.082
MR Volume (TEE), ml [mean ± SD]	70± 33	50 ± 31	0.030
MR V_max_ (TEE), m/s [mean ± SD]	5 ± 1	5 ± 1	0.085
Mitral Valve Orifice Area, cm^2^ [mean ± SD]	4.9 ± 2.1	4.8 ± 1.6	0.80
MV MPG, mmHg [mean ± SD]	4.0 ± 1.5	2.2 ± 1.1	<0.001
Annular Area (measured in 3D), cm^2^ [mean ± SD]	12.2 ± 3.5	11.5 ± 3.2	0.40
Annular Circumference, cm [mean ± SD]	12.4 ± 1.8	12.1 ± 1.7	0.40
Aortomitral Angle, ° [mean ± SD]	135 ± 12	139 ± 14	0.30
Tenting Height, cm [mean ± SD]	0.4 ± 0.2	0.6 ± 0.4	0.020
Tenting Area, cm^2^ [mean ± SD]	1.0 ± 0.7	1.9 ± 1.5	0.004
Tenting Volume, mL [mean ± SD]	1.7 ± 1.2	2.6 ± 1.9	0.059
Anterior Billowing Height, mm [mean ± SD]	0.6± 1.0	0.1 ± 0.4	<0.001
Posterior Billowing Height, mm [mean ± SD]	2.2 ± 2.7	1.0 ± 1.8	0.023

Table Legend: EROA = Effective Regurgitant Orifice Area; IQR = interquartile range; LA = left atrium; LVEF = left ventricular ejection fraction; LVEDD = left ventricular end-diastolic diameter; LVEDV = left ventricular end-diastolic volume; LVESD = left ventricular end-systolic diameter; LVESV = left ventricular end-systolic volume; MPG = mean pressure gradient; MR = mitral regurgitation; M-TEER = mitral transcatheter edge-to-edge repair; MV = mitral valve; RA = right atrium; RV = right ventricle; SD = standard deviation; TAPSE = tricuspid annular plane systolic excursion; TEE = transesophageal echocardiography. ^1^ Wilcoxon rank test, Kruskal–Wallis test, Fisher’s exact test, or Pearson’s chi-square test, as appropriate.

**Table 3:** Baseline demographic and clinical characteristics among patients evaluated for TMVR.

	TMVR Refused	TMVR Accepted	*p*-Value ^1^
Characteristic	*n* = 77	*n* = 27	
Age, years [median (IQR)]	80 (76.5–83)	79 (72–83)	0.70
Female [*n* (%)]	40 (53)	11 (41)	0.30
BMI, kg/m^2^ [mean ± SD]	25.8 ± 4.3	25.6 ± 3.2	0.70
STS Score, % [median (IQR)]	2.7 (1.5–3.4)	3.7 (2.8–6.5)	0.017
EuroSCORE II, % [median (IQR)]	4.5 (2.7–9.0)	6.6 (4.7–12.1)	0.041
Atrial fibrillation [*n* (%)]	56 (74)	22 (81)	0.40
Prior MI [*n* (%)]	10 (13)	5 (19)	0.05
Prior cardiac surgery [*n* (%)]	57 (75)	25 (93)	0.05
NYHA class baseline ≥ 3 [*n* (%)]	65 (84)	21 (78)	0.60
Prior MV intervention			0.80
None [*n* (%)]	68 (88)	26 (96)	
Prior MV replacement [*n* (%)]	2 (3)	0	
Prior MV repair [*n* (%)]	7 (9)	1 (4)	
MV disease			0.70
MV regurgitation [*n* (%)]	49 (64)	16 (59)	
Combined MV disease [*n* (%)]	27 (35)	9 (41)	
MV Stenosis [*n* (%)]	1 (1)	0 (0)	
MV regurgitation etiology			0.20
Degenerative [*n* (%)]	37 (48)	11 (41)	
Functional [*n* (%)]	21 (27)	13 (48)	
Mixed [*n* (%)]	14 (18)	2 (7)	
Residual [*n* (%)]	5 (7)	1 (4)	
MR severity			0.70
Mild [*n* (%)]	1 (1.5)	0 (0)	
Mild to moderate [*n* (%)]	1 (1.5)	0 (0)	
Moderate to severe [*n* (%)]	21 (27)	5 (19)	
Severe [*n* (%)]	54 (70)	22 (81)	

Table Legend: BMI = Body Mass Index; EuroSCORE II = European System for Cardiac Operative Risk Evaluation Score II; IQR = interquartile range; MI = myocardial infarction; MR = mitral regurgitation; MV = mitral valve; NYHA = New York Heart Association; STS Score = Society of Thoracic Surgeons Risk Score; TMVR = transcatheter mitral valve replacement. ^1^ Wilcoxon rank test, Kruskal–Wallis test, Fisher’s exact test, or Pearson’s chi-square test, as appropriate.

**Table 4:** Baseline echocardiographic characteristics among patients evaluated for TMVR.

	TMVR Refused	TMVR Accepted	*p*-Value ^1^
Characteristic	*n* = 77	*n* = 27	
LVEF, % [mean ± SD]	54 ± 10	49 ± 9	0.014
LVEDD, mm [median (IQR)]	53 (46–59)	57 (53–62)	0.027
LVEDV, ml [median (IQR)]	95 (74–141)	111 (85–134)	0.40
LVESD, mm [median (IQR)]	37 (30–46)	41 (36–50)	0.014
LVESV, ml [median (IQR)]	42 (29–70)	53 (42–76)	0.046
LA Diameter, mm [median (IQR)]	49 (43–58)	55 (50–59)	0.07
LA Volume, ml [mean ± SD]	142 ± 76	141 ± 57	0.50
RA Area, cm^2^ [mean ± SD]	24 ± 11	26 ± 10	0.30
RV Basal Diameter, mm [mean ± SD]	35 ± 10	38 ± 12	0.10
TAPSE, mm [mean ± SD]	18 ± 5	17 ± 4	0.50
MR EROA, cm^2^ [mean ± SD]	0.5 ± 0.4	0.5 ± 0.2	0.20
MR Volume, ml [mean ± SD]	62 ± 24	71 ± 24	0.20
MR V_max_, m/s [mean ± SD]	5 ± 1	5 ± 1	0.50
MR EROA (TEE), mm [mean ± SD]	0.6 ± 1.0	0.5 ± 0.1	0.60
MR Volume (TEE), ml [mean ± SD]	57 ± 26	65 ± 23	0.20
MR V_max_ (TEE), m/s [mean ± SD]	5 ± 1	5 ± 1	0.60
Mitral Valve Orifice Area, cm^2^ [mean ± SD]	3.6 ± 1.6	3.6 ± 2.1	0.70
MV MPG, mmHg [mean ± SD]	4.8 ± 3.6	3.8 ± 2.5	0.20
Annular Area (measured in 3D), cm^2^ [mean ± SD]	14.1 ± 4.2	15.0 ± 3.7	0.30
Annular Circumference, cm [mean ± SD]	13.7 ± 2.1	14.1 ± 1.9	0.30
Aortomitral Angle, ° [mean ± SD]	120 ± 17	121 ± 19	>0.90
Tenting Height, cm [mean ± SD]	0.5 ± 0.3	0.7 ± 0.4	0.021
Tenting Area, cm^2^ [mean ± SD]	1.5 ± 0.9	2.6 ± 2.5	0.004
Tenting Volume, mL [mean ± SD]	3.4 ± 3.1	5.5 ± 4.2	0.006
Anterior Billowing Height, mm [mean ± SD]	0.5 ± 1.3	0.4 ± 1.1	0.90
Posterior Billowing Height, mm [mean ± SD]	0.9 ± 1.5	0.2 ± 0.5	0.08

Table Legend: EROA = Effective Regurgitant Orifice Area; IQR = interquartile range; LA = left atrium; LVEF = left ventricular ejection fraction; LVEDD = left ventricular end-diastolic diameter; LVEDV = left ventricular end-diastolic volume; LVESD = left ventricular end-systolic diameter; LVESV = left ventricular end-systolic volume; MPG = mean pressure gradient; MR = mitral regurgitation; MV = mitral valve; RA = right atrium; RV = right ventricle; SD = standard deviation; TAPSE = tricuspid annular plane systolic excursion; TEE = transesophageal echocardiography; TMVR = transcatheter mitral valve replacement. ^1^ Wilcoxon rank test, Kruskal–Wallis test, Fisher’s exact test, or Pearson’s chi-square test, as appropriate.

**Table 5:** Baseline demographic and clinical characteristics among patients evaluated for M-TEER and TMVR.

Characteristic	M-TEER Screened	TMVR Screened	*p*-Value ^1^
	*n* = 189	*n* = 104	
Age, years [median (IQR)]	79 (73–83)	80 (76–83)	0.20
Female [*n* (%)]	80 (42%)	51(50%)	0.20
BMI, kg/m^2^ [mean ± SD]	26.5 ± 6	25.8 ± 4	0.90
STS Score, % [median (IQR)]	2.4 (1.3–4.1)	2.8 (1.7–4.8)	0.14
EuroSCORE II, % [median (IQR)]	4.3 (2.5–7.3)	5.1 (3.1–9.1)	0.049
Atrial fibrillation [*n* (%)]	124 (66%)	78 (76%)	0.74
Prior MI [*n* (%)]	31 (16%)	15 (15%)	0.70
Prior cardiac surgery [*n* (%)]	42 (22%)	82 (80%)	<0.001
NYHA class baseline ≥ 3 [*n* (%)]	159 (85%)	83 (82%)	0.50
Prior MV intervention			0.11
None [*n* (%)]	181 (96%)	93 (90%)	
Prior MV replacement [*n* (%)]	2 (1.1%)	2 (1.9%)	
Prior MV repair [*n* (%)]	6 (3.2%)	8 (7.8%)	
MV disease			<0.001
MV regurgitation [*n* (%)]	181 (96%)	65 (63%)	
Combined MV disease [*n* (%)]	8 (4.2%)	38 (37%)	
MV stenosis [*n* (%)]	0 (0%)	1 (1%)	
MV regurgitation etiology			<0.001
Degenerative [*n* (%)]	68 (36%)	48 (46%)	
Functional [*n* (%)]	100 (53%)	34 (33%)	
Mixed [*n* (%)]	21 (11%)	16 (15%)	
Residual [*n* (%)]	0 (0%)	6 (5.8%)	
MR severity			<0.001
Mild [*n* (%)]	1 (0.5%)	1 (1%)	
Mild to moderate [*n* (%)]	4 (2.1%)	1 (1%)	
Moderate to severe [*n* (%)]	90 (48%)	26 (25%)	
Severe [*n* (%)]	94 (50%)	76 (73%)	

Table Legend: BMI = Body Mass Index; EuroSCORE II = European System for Cardiac Operative Risk Evaluation Score II; IQR = interquartile range; MI = myocardial infarction; MR = mitral regurgitation; M-TEER = mitral transcatheter edge-to-edge repair; MV = mitral valve; NYHA = New York Heart Association; STS Score = Society of Thoracic Surgeons Risk Score; TMVR = transcatheter mitral valve replacement. ^1^ Wilcoxon rank test, Kruskal–Wallis test, Fisher’s exact test, or Pearson’s chi-square test, as appropriate.

**Table 6:** Echocardiographic parameters of patients screened for M-TEER and TMVR.

Characteristic	M-TEER Screened	TMVR Screened	*p*-Value ^1^
	*n* = 189	*n* = 104	
LVEF, % [mean ± SD]	50 ± 15	52 ± 10	0.50
LVEDD, mm [median (IQR)]	57 (51–63)	55 (48–60)	0.041
LVEDV, ml [median (IQR)]	104 (72–154)	101 (75–137)	0.70
LVESD, mm [median (IQR)]	39 (33–50)	39 (32–48)	0.40
LVESV, ml [median (IQR)]	47 (30–84)	50 (32–72)	0.90
LA Diameter, mm [median (IQR)]	52 (46–58)	52 (45–58)	0.90
LA Volume, mL [mean ± SD]	52 ± 10	52 ± 10	0.90
RA Area, cm^2^ [mean ± SD]	24 ± 10	24 ± 11	0.80
RV Basal Diameter, mm [mean ± SD]	37 ± 10	36 ± 11	0.12
TAPSE, mm [mean ± SD]	19 ± 5	18 ± 5	0.028
MR EROA, cm^2^ [mean ± SD]	0.4 ± 0.3	0.5 ± 0.3	0.001
MR Volume, ml [mean ± SD]	58 ± 34	64 ± 24	0.003
MR V_max_, m/s [mean ± SD]	5.3 ± 1	5.0 ± 1	0.06
MR EROA (TEE), mm [mean ± SD]	0.4 ± 0.3	0.6 ± 1	<0.001
MR Volume (TEE), ml [mean ± SD]	53 ± 32	59 ± 25	0.017
MR V_max_ (TEE), m/s [mean ± SD]	5 ± 1	4.7 ± 1	0.035
Mitral Valve Orifice Area, cm^2^ [mean ± SD]	4.8 ± 2	3.6 ± 2	<0.001
MV MPG, mmHg [mean ± SD]	2.4 ± 1	4.6 ± 3	<0.001
Annular Area (measured in 3D), cm^2^ [mean ± SD]	12 ± 3	15 ± 4	<0.001
Annular Circumference, cm [mean ± SD]	12 ± 2	14 ± 2	<0.001
Aortomitral Angle, ° [mean ± SD]	140 ± 13	120 ± 17	<0.001
Tenting Height, cm [mean ± SD]	0.6 ± 0.4	0.6 ± 0.3	0.20
Tenting Area, cm^2^ [mean ± SD]	1.8 ± 1.4	1.8 ± 1.6	0.80
Tenting Volume, mL [mean ± SD]	2.5 ± 2	4.0 ± 4	<0.001
Anterior Billowing Height, mm [mean ± SD]	0.1 ± 0.5	0.6 ± 1	0.007
Posterior Billowing Height, mm [mean ± SD]	1.1 ± 2	0.7 ± 1	0.30

Table Legend: BMI = Body Mass Index; EuroSCORE II = European System for Cardiac Operative Risk Evaluation Score II; IQR = interquartile range; MI = myocardial infarction; MR = mitral regurgitation; M-TEER = mitral transcatheter edge-to-edge repair; MV = mitral valve; NYHA = New York Heart Association; STS Score = Society of Thoracic Surgeons Risk Score; TMVR = transcatheter mitral valve replacement. ^1^ Wilcoxon rank test, Kruskal–Wallis test, Fisher’s exact test, or Pearson’s chi-square test, as appropriate.

## Data Availability

The original contributions presented in this study are included in the article/Supplementary Material. Further inquiries can be directed at the corresponding author.

## Supplementary Materials

The following supporting information can be downloaded at: https://www.mdpi.com/article/10.3390/jcm14207275/s1, Table S1: Baseline Demographic and Clinical Characteristics Among Patients Rejected for M-TEER or TMVR; Table S2: Echocardiographic parameters of patients rejected for M-TEER or TMVR; Table S3: Baseline Demographic and Clinical Characteristics Among Patients Accepted for M-TEER or TMVR; Table S4: Baseline echocardiographic parameters of patients accepted for M-TEER or TMVR.

## Abbreviations

The following abbreviations are used in this manuscript:

BMI	Body Mass Index
CE	Conformité Européenne
DMR	Degenerative mitral regurgitation
FMR	Functional mitral regurgitation
LA	Left atrium
LVEDD	Left ventricular enddiastolic diameter
LVEF	Left ventricular ejection fraction
LVESD	left ventricular endsystolic diameter
LVESV	left ventricular endsystolic volume
LVOTO	Left ventricular outflow tract obstruction
MPG	Mean pressure gradient
MR	Mitral regurgitation
M-TEER	Mitral transcatheter edge-to-edge repair
M-TEER-A	Patients accepted for Mitral transcatheter edge-to-edge repair
M-TEER-R	Patients rejected for Mitral transcatheter edge-to-edge repair
MVARC	Mitral Valve Academic Research Consortium
MVQ	Mitral valve quantification
NYHA	New York Heart Association
PVL	Paravalvular leakage
SAM	Systolic anterior motion
TAVR	Transcatheter aortic valve replacement
TMVR	Transcatheter mitral valve replacement
TMVR-A	Patients accepted for Transcatheter mitral valve replacement
TMVR-R	Patients rejected for Transcatheter mitral valve replacement
TEE	Transesophageal echocardiography
TTE	Transthoracic echocardiography
VC	Vena contracta
