# Supplementary material for: Characterization of Patients Unsuited for Transcatheter Mitral Valve Interventions
Source: J Clin Med. 2025 Oct 15;14(20):7275. doi: 10.3390/jcm14207275 (PMC12564858; doi:10.3390/jcm14207275)
Supplement: Supplementary file 1 [file jcm-14-07275-s001.zip › jcm-3897506-supplementary.pdf]

Journal of Clinical Medicine

Supplemental Material:

Table S1: Baseline Demographic and Clinical Characteristics Among Patients Rejected for M-TEER or TMVR

Characteristic	M-TEER Refused $n = 21$	TMVR Refused $n = 77$	*P*-Value[1]
Age, years [median (IQR)]	75 (70-79)	80 (77-83)	0.002
Female [n (%)]	6 (29%)	40 (53%)	0.051
BMI, kg/m² [mean ± SD]	27±8	26±4	0.50
STS Score, % [median (IQR)]	1.3 (0.8-2.7)	2.7 (1.5-3.4)	0.014
EuroSCORE II, % [median (IQR)]	2.3 (1.5-3.5)	4.5 (2.7-9.0)	0.008
Atrial Fibrillation [n (%)]	15 (71%)	56 (74%)	0.80
Prior MI [n (%)]	4 (19%)	10 (13%)	0.50
Prior cardiac surgery [n (%)]	4 (19%)	57 (75%)	<0.001
NYHA class baseline ≥3 [n (%)]	17 (81%)	62 (84%)	0.70
**prior MV intervention**			0.40
None [n (%)]	17 (81%)	67 (88%)	
Prior MV replacement [n (%)]	2 (9.5%)	2 (2.6%)	
Prior MV repair [n (%)]	2 (9.5%)	7 (9.2%)	
**MV disease**			0.034
MV regurgitation [n (%)]	19 (90%)	49 (64%)	
Combined MV disease [n (%)]	2 (9.5%)	27 (35%)	
MV Stenosis [n (%)]	0 (0%)	1 (1.3%)	
**MV regurgitation etiology**			0.60
Degenerative [n (%)]	12 (57%)	37 (77%)	
Functional [n (%)]	4 (19%)	21 (27%)	
Mixed [n (%)]	5 (24%)	14 (18%)	
Residual [n (%)]	0 (0%)	5 (6.5%)	
**MR severity**			0.30
Mild [n (%)]	1 (4.8%)	1 (1.3%)	
Mild-to-moderate [n (%)]	0 (0%)	1 (1.3%)	
Moderate-to-severe [n (%)]	9 (43%)	21 (27%)	
Severe [n (%)]	11 (52%)	54 (70%)	

Table Legend: BMI = Body-Mass Index, EuroSCORE II = European System for Cardiac Operative Risk Evaluation Score II, IQR = Interquartile Range, MI = Myocardial Infarction, MR = Mitral Regurgitation, M-TEER = Mitral Transcatheter Edge-to-Edge Repair, MV = Mitral Valve, NYHA = New York Heart Association, STS-Score = Society of Thoracic Surgeons Risk Score. [1]Wilcoxon Rank Test, Kruskal Wallis Test, Fisher's exact test or Pearson Chi square test, as appropriate.

Table S2: Echocardiographic parameters of patients rejected for M-TEER or TMVR

**Characteristic**	**M-TEER Refused** *n* = 21	**TMVR Refused** *n* = 77	*P*-Value[1]
LVEF, % [mean ± SD]	57±16	54±10	0.20
LVEDD, mm [median (IQR)]	56 (51-61)	53 (46-59)	0.20
LVEDV, ml [median (IQR)]	113 (84-143)	95 (74-141)	0.50
LVESD, mm [median (IQR)]	36 (32-44)	37 (30-46)	>0.90
LVESV, ml [median (IQR)]	48 (23-69)	42 (29-70)	0.70
LA Diameter, mm [median (IQR)]	48 (39-55)	49 (43-58)	0.30
LA Volume, mL [mean ± SD]	131±99	142±76	0.20
RA Area, cm^2 [mean ± SD]	21±10	24±11	0.20
RV Basal Diameter, mm [mean ± SD]	33±10	35±11	0.20
TAPSE, mm [mean ± SD]	19±4	18±5	0.30
MR EROA, cm^2 [mean ± SD]	0.4±0.3	0.5±0.4	0.035
MR Regurgitation Volume, ml [mean ± SD]	58±28	62±24	0.20
MR V_{max}, m/s [mean ± SD]	5.8±0.8	5.0±1.1	0.004
MR EROA (TEE), mm [mean ± SD]	0.4±0.2	0.6±1.1	0.50
MR Regurgitation Volume (TEE), ml [mean ± SD]	70±33	57±26	0.20
MR V_{max} (TEE), m/s [mean ± SD]	5.4±0.7	4.8±0.9	0.019
Mitral Valve Orifice Area, cm^2 [mean ± SD]	4.9±2	3.6±2	0.014
MV MPG, mmHg [mean ± SD]	4.0±2	4.8±4	>0.90
Annular Area (measured in 3D), cm^2 [mean ± SD]	12±3	15±4	0.026
Annular Circumference, cm [mean ± SD]	12±2	14±2	0.023
Aortomitral Angle, ° [mean ± SD]	135±12	120±16	0.001
Tenting Height, cm [mean ± SD]	0.4±0.2	0.5±0.3	0.30
Tenting Area, cm^2 [mean ± SD]	1.0±0.7	1.9±1.8	0.018
Tenting Volume, mL [mean ± SD]	1.7±1.2	4.1±3.9	0.002
Anterior Billowing Height, mm [mean ± SD]	0.6±1	0.6±1	0.30
Posterior Billowing Height, mm [mean ± SD]	2.2±3	0.8±1	0.023

Table Legend: BMI = Body-Mass Index, EuroSCORE II = European System for Cardiac Operative Risk Evaluation Score II, IQR = Interquartile Range, MI = Myocardial Infarction, MR = Mitral Regurgitation, M-TEER = Mitral Transcatheter Edge-to-Edge Repair, MV = Mitral Valve, NYHA = New York Heart Association, STS-Score = Society of Thoracic Surgeons Risk Score, TMVR= Transcatheter Mitral Valve Replacement. [1]Wilcoxon Rank Test, Kruskal Wallis Test, Fisher's exact test or Pearson Chi square test, as appropriate.

Table S3: Baseline Demographic and Clinical Characteristics Among Patients Accepted for M-TEER or TMVR.

Characteristic	M-TEER Accepted n = 168	TMVR Accepted n = 27	P-Value[1]
Age, years [median (IQR)]	80 (74-83)	79 (72-83)	>0.90
Female [n (%)]	74 (44%)	11 (41%)	0.70
BMI, kg/m² [mean ± SD]	26±6	26±3	0.80
STS Score, % [median (IQR)]	2.6 (1.5-4.6)	3.7 (2.8-6.5)	0.034
EuroSCORE II, % [median (IQR)]	4.5 (2.7-7.4)	6.6 (4.7-12.1)	0.012
Atrial Fibrillation [n (%)]	109 (65%)	22 (81%)	0.09
Prior MI [n (%)]	27 (16%)	5 (19%)	0.80
Prior cardiac surgery [n (%)]	38 (23%)	25 (93%)	<0.001
NYHA class baseline ≥3 [n (%)]	142 (86%)	21 (78%)	0.40
**prior MV intervention**			0.50
None [n (%)]	164 (98%)	26 (96%)	
Prior MV replacement [n (%)]	0 (0%)	0 (0%)	
Prior MV repair [n (%)]	4 (2%)	1 (4%)	
**MV disease**			<0.001
MV regurgitation [n (%)]	162 (96%)	16 (59%)	
Combined MV disease [n (%)]	6 (4%)	11 (41%)	
MV Stenosis [n (%)]	0 (0%)	0 (0%)	
**MV regurgitation etiology**			0.20
Degenerative [n (%)]	56 (33%)	11 (41%)	
Functional [n (%)]	96 (57%)	13 (48%)	
Mixed [n (%)]	16 (10%)	2 (7%)	
Residual [n (%)]	0 (0%)	1 (4%)	
**MR severity**			0.007
Mild [n (%)]	0 (0%)	0 (0%)	
Mild-to-moderate [n (%)]	4 (3%)	0 (0%)	
Moderate-to-severe [n (%)]	81 (48%)	5 (19%)	
Severe [n (%)]	83 (49%)	22 (81%)	

Table Legend: BMI = Body-Mass Index, EuroSCORE II = European System for Cardiac Operative Risk Evaluation Score II, IQR = Interquartile Range, MI = Myocardial Infarction, MR = Mitral Regurgitation, M-TEER = Mitral Transcatheter Edge-to-Edge Repair, MV = Mitral Valve, NYHA = New York Heart Association, STS-Score = Society of Thoracic Surgeons Risk Score. [1]Wilcoxon Rank Test, Kruskal Wallis Test, Fisher's exact test or Pearson Chi square test, as appropriate.

Table S4: Baseline echocardiographic parameters of patients accepted for M-TEER or TMVR

Characteristic	M-TEER Accepted n = 168	TMVR Accepted n = 27	P-Value[1]
LVEF, % [mean ± SD]	50±15	49±9	0.40
LVEDD, mm [median (IQR)]	57 (51-63)	57 (53-62)	0.70
LVEDV, ml [median (IQR)]	100 (71-158)	111 (85-134)	0.60
LVESD, mm [median (IQR)]	39 (33-50)	41 (36-50)	0.20
LVESV, ml [median (IQR)]	47 (31-87)	53 (42-76)	0.15
LA Diameter, mm [median (IQR)]	52 (46-59)	55 (50-59)	0.20

**Characteristic**	**M-TEER Accepted** *n* = 168	**TMVR Accepted** *n* = 27	*P*-Value[1]
LA Volume, mL [mean ± SD]	127±61	141±57	0.20
RA Area, cm^2 [mean ± SD]	24±10	26±10	0.50
RV Basal Diameter, mm [mean ± SD]	38±9	38±12	0.80
TAPSE, mm [mean ± SD]	19±5	17±4	0.05
MR EROA, cm^2 [mean ± SD]	0.4±0.3	0.5±0.2	0.017
MR Regurgitation Volume, ml [mean ± SD]	58±34	71±24	0.007
MR V_{max}, m/s [mean ± SD]	5.2±1	4.9±1	0.20
MR EROA (TEE), mm [mean ± SD]	0.4±0.3	0.5±0.1	0.001
MR Regurgitation Volume (TEE), ml [mean ± SD]	50±31	65±23	0.006
MR V_{max} (TEE), m/s [mean ± SD]	5.0±1	4.6±1	0.10
Mitral Valve Orifice Area, cm^2 [mean ± SD]	4.8±2	3.6±2	0.001
MV MPG, mmHg [mean ± SD]	2.2±1	3.8±2	<0.001
Annular Area (measured in 3D), cm^2 [mean ± SD]	12±3	15±5	<0.001
Annular Circumference, cm [mean ± SD]	12±2	14±2	<0.001
Aortomitral Angle, ° [mean ± SD]	139±14	120±21	<0.001
Tenting Height, cm [mean ± SD]	0.6±0.4	0.6±0.3	>0.90
Tenting Area, cm^2 [mean ± SD]	1.9±1.5	1.7±0.7	0.70
Tenting Volume, mL [mean ± SD]	2.6±1.9	3.4±1.7	0.021
Anterior Billowing Height, mm [mean ± SD]	0.1±0.4	0.4±1.1	0.046
Posterior Billowing Height, mm [mean ± SD]	1.0±1.8	0.5±1.1	0.20

Table Legend: BMI = Body-Mass Index, EuroSCORE II = European System for Cardiac Operative Risk Evaluation Score II, IQR = Interquartile Range, MI = Myocardial Infarction, MR = Mitral Regurgitation, M-TEER = Mitral Transcatheter Edge-to-Edge Repair, MV = Mitral Valve, NYHA = New York Heart Association, STS-Score = Society of Thoracic Surgeons Risk Score, TMVR= Transcatheter Mitral Valve Replacement. [1]Wilcoxon Rank Test, Kruskal Wallis Test, Fisher's exact test or Pearson Chi square test, as appropriate.
